# Supplementary material for: Branch error reduction criterion-based signal recursive decomposition and its application to wind power generation forecasting
Source: PLoS One. 2024 Mar 22;19(3):e0299955. doi: 10.1371/journal.pone.0299955 (PMC10959340; doi:10.1371/journal.pone.0299955)
Supplement: Attachment — Submitted filename: Response to Reviewers.docx [file pone.0299955.s002.docx]

**Main changes in the revised version are highlighted in red.**

A list of changes in the revised manuscript is as follows.

**List of Actions：**

**LOA1**: References have been supplemented in the introduction section and the comparative experiments section according to the review’s kind suggestions.

**LOA2**: The citations formats have been corrected in the third paragraph of the introduction section (Page 2).

**LOA3**: The reason for modal decomposition of time series has been supplemented in the forth paragraph of the introduction section (Line 37-43).

**LOA4**: ADF test is supplemented in Table 2 (Page 7) to prove the non-stationarity of the original time series used in the experimental section.

The responses to the comments of academic editor and reviewers are as the following:

**To Academic Editor**

Thank you so much for your professional and valuable comments on our manuscripts, we have made changes and improvements accordingly.

**Comments:**

1. Please ensure that your manuscript meets PLOS ONE's style requirements, including those for file naming.

**Reply**: Thank you so much for the kind suggestion. We have carefully checked our manuscript to ensure that this manuscript meets PLOS ONE's style requirements.

2. Please note that PLOS ONE has specific guidelines on code sharing for submissions in which author-generated code underpins the findings in the manuscript. In these cases, all author-generated code must be made available without restrictions upon publication of the work. Please review our guidelines at https://journals.plos.org/plosone/s/materials-and-software-sharing#loc-sharing-code and ensure that your code is shared in a way that follows best practice and facilitates reproducibility and reuse.

**Reply**: Thank you very much for your reminder. The code has been packaged for easy running.

3. The Funding Statement and Competing Interests Statement need to be updated.

**Reply**: Thank you for your kind suggestion. The Funding Statement and Competing Interests Statement are updated.

4. Funding information should not appear in the Acknowledgments section or other areas of your manuscript. We will only publish funding information present in the Funding Statement section of the online submission form. Please include your amended statements within your cover letter; we will change the online submission form on your behalf.

**Reply**: Sorry for the oversight, the funding information has been placed in Funding Statement section of the online submission form.

The updated Funding Statement is as the following:

Funding information: State Grid Fujian Electric Power Co. Ltd.: SGTYHT/20-JS-223(SGFJJY00GHJS2200054). The funders had no role in study design, data collection and analysis, decision to publish, or preparation of the manuscript. The funder provided support in the form of salaries for authors Fen Xiao and Siyu Yang, but did not have any additional role in the study design, data collection and analysis, decision to publish, or preparation of the manuscript. The specific roles of these authors are articulated in the ‘author contributions’ section.

5. In your Data Availability statement, you have not specified where the minimal data set underlying the results described in your manuscript can be found.

**Reply**：Thank you for your suggestion. The minimal data set used in the manuscript has been uploaded as Supporting Information.

6. Please review your reference list to ensure that it is complete and correct.

**Reply**: Thank you for the kind suggestion. All references have been checked carefully. Four new references have been supplemented.

**To Reviewers #1**

We really appreciate the professional and valuable comments on our manuscript. We have made the revisions and improvements accordingly.

1. In line 18 its written In Ref. [6] which i presume was supposed to write the author's name like what the authors did in line 19. This needs correction. Similar mistake is made in line 24 and 43.

**Reply**: Thank you for the kind suggestion. The citations formats have been corrected accordingly.

2. Decomposition has been used as a preprocessing method in many studies as also cited in this paper. I suggest adding one or 2 sentences after paragraph 1 in Introduction justifying why the wind generation data needs to be decomposed.

**Reply**: Thank you for the professional comment. The reason for the decomposition of the generation time series is supplemented at the beginning of the 4th paragraph in the introduction section: “The time series of electricity generation is generally a broadband signal, and its future trend is not stable. Therefore, it is difficult to approximate the relationship between historical measurements and its future changes. The future trend of a narrowband signal is normally considered to be more stable. Therefore, the second type of hybrid model is used to decompose the time series of power generation into narrowband modes, and the final forecasted results are obtained by summarizing the forecasted results of each mode” (Line 37-43).

3. In Line 192 I suggest carrying out stationarity Tests such as the Augmented Dickey-Fuller (ADF) test and the Kwiatkowski-Phillips-Schmidt-Shin (KPSS) test and then make a conclusion that the data is non-stationary.

**Reply**: Thank you for the enlightening comment.

The ADF test results of partial and total generation time series of 2020 and 2021 are supplemented to Table 2 in the revised manuscript. Detailed statistics of the ADF test are also presented in Table R.1, which indicates the non-stationarity of the time series data. Due to limited space, can we just not show Table R.1 in the revised manuscript, thank you.

Table R.1. ADF test results and critical values

|  | Season | ADF test results | Critical values | | |
| --- | --- | --- | --- | --- | --- |
|  |  |  | 1% | 5% | 10% |
| 2020 | A year | -1.5399 | -3.4489 | -2.8697 | -2.5711 |
|  | Spring | -2.6172 | -3.5117 | -2.8970 | -2.5857 |
|  | Summer | -1.8411 | -3.5069 | -2.8950 | -2.5846 |
|  | Autumn | 2.7401 | -3.5117 | -2.8970 | -2.5857 |
|  | Winter | -1.6443 | -3.5171 | -2.8994 | -2.5870 |
| 2021 | A year | -2.0990 | -3.4489 | -2.8697 | -2.5711 |
|  | Spring | -2.4970 | -3.5127 | -2.8975 | -2.5859 |
|  | Summer | -3.2289 | -3.5061 | -2.8946 | -2.5844 |
|  | Autumn | -1.8297 | -3.5043 | -2.8939 | -2.5840 |
|  | Winter | -2.2927 | -3.5149 | -2.8984 | -2.5864 |

4. It would be interesting to add few more years instead of using only 2 years to carry out a study.

**Reply:** Thank you for the practically significant suggestion.

The historical data of power generation in Fujian Province from Jan 1 2012 to Jun 30 2022 is shown in Fig. R.1, with a total of 3834 daily power generation data. A total of 3653 power generation data in the decade 2012- 2021 is selected as the training set, and a total of 181 power generation data in the first six months of 2022 is selected as the testing set. The same comparison experiments are conducted against the manuscript and the results are shown in Table R.2.

Fig. R.1 Power generation trend of Fujian Province from Jan o1 2012 to Jun 30 2019.

Table R.2. Comparative experiments

| Indicator | Model | First Level | Group A | Group B | Group C | Group D |
| --- | --- | --- | --- | --- | --- | --- |
| MAE | LSSVM | 988.4257 | 523.8940 | 544.3286 | 522.9598 | 374.2315 |
|  | ELM | 711.1507 | 1061.5339 | 655.0425 | 584.3203 | 556.2979 |
|  | DBN | 727.5372 | 1194.8209 | 1185.0920 | 743.8101 | 381.7586 |
|  | LSTM | 1304.2324 | 1542.0887 | 1623.0941 | 815.9724 | 792.8939 |
| RMSE | LSSVM | 1681.6103 | 884.4244 | 927.3799 | 880.8208 | 655.9868 |
|  | ELM | 924.8178 | 3255.6449 | 663.0800 | 933.0626 | 889.2999 |
|  | DBN | 934.8419 | 1337.8516 | 1296.2681 | 887.8033 | 500.3471 |
|  | LSTM | 1616.9842 | 1782.9000 | 1822.7882 | 1048.3438 | 987.1922 |
| MAPE  （%） | LSSVM | 1.2973 | 0.6950 | 0.7090 | 0.6863 | 0.4917 |
|  | ELM | 0.9581 | 1.3540 | 0.8425 | 0.7797 | 0.7299 |
|  | DBN | 0.9775 | 1.5267 | 1.5155 | 0.9502 | 0.5037 |
|  | LSTM | 1.7752 | 2.0279 | 2.1465 | 1.1259 | 1.0895 |

According to Table R. 2, the same conclusions can be drawn:

- More decomposition layers or more decomposition numbers cannot be equated with better decomposition effect;
- There are randomness and limitations in the final effect of direct decomposition and decomposition according to whether the center frequency is aliased;
- Recursive decomposition based on BER performs more consistently and efficiently in the experiment.

Because the trend of power generation and the influencing factors change over time, the distribution of the dataset used for training is not consistent with the new data, resulting in the previous model not being able to forecast the present data at a high level of accuracy, which indicates that the distribution drift occurs.

Although the same conclusions can be drawn, the electricity generation data have several characteristics: they are highly influenced by policy, data distribution evolves over time. Compared with one year's data, too much experimental data seems to be less suitable for comparison and presentation to some extent. Therefore, would it be more succinct to use only single year data in the experiment? We look forward to your valuable suggestions.

**To Reviewers #2**

The authors would like to thank you for taking the time to read our manuscript. We have modified our manuscript according to the suggestions and comments.

1. Some statements in the introductory section need to be cited.

**Reply:** Thank you for the kind suggestion. In the introduction section, three references are supplemented, i.e., Ref [1], Ref [2] and Ref [4]. In the comparative experiments section, Ref [25] is supplemented.

Once again, Thanks all of you so much for the valuable comments, suggestions and help.
